# Fetal Alcohol Spectrum Disorders: Awareness to Insight in Just 50 Years

**DOI:** 10.35946/arcr.v42.1.05

**Published:** 2022-02-24

**Authors:** Michael E. Charness

**Affiliations:** VA Boston Healthcare System, Boston, Massachusetts. Harvard Medical School, Boston, Massachusetts. Boston University School of Medicine, Boston, Massachusetts

**Keywords:** fetal alcohol syndrome, fetal alcohol spectrum disorders, alcohol, brain development, craniofacial dysmorphology

## Abstract

This article is part of a Festschrift commemorating the 50th anniversary of the National Institute on Alcohol Abuse and Alcoholism (NIAAA). Established in 1970, first as part of the National Institute of Mental Health and later as an independent institute of the National Institutes of Health, NIAAA today is the world’s largest funding agency for alcohol research. In addition to its own intramural research program, NIAAA supports the entire spectrum of innovative basic, translational, and clinical research to advance the diagnosis, prevention, and treatment of alcohol use disorder and alcohol-related problems. To celebrate the anniversary, NIAAA hosted a 2-day symposium, “Alcohol Across the Lifespan: 50 Years of Evidence-Based Diagnosis, Prevention, and Treatment Research,” devoted to key topics within the field of alcohol research. This article is based on Dr. Charness’ presentation at the event. NIAAA Director George F. Koob, Ph.D., serves as editor of the Festschrift.

The establishment of the National Institute on Alcohol Abuse and Alcoholism (NIAAA) in 1971 was bracketed by three seminal papers that laid the groundwork for the field of fetal alcohol spectrum disorders (FASD) research. In 1968, Lemoine et al.^1^ described children with birth defects and neurodevelopmental disorders associated with prenatal alcohol exposure (PAE). This French-language report was not widely appreciated until after the publication in 1973 of two landmark papers in *The Lancet*,^2^,^3^ providing the first English-language description of fetal alcohol syndrome (FAS). The subsequent recognition of the high global prevalence of FASD and FAS highlighted a paradox. If alcohol and PAE have been ubiquitous since antiquity, why was FASD not recognized sooner?

Indeed, there were hints dating back to biblical times that PAE was harmful to the developing fetus (reviewed by Jones and Smith^2^ and by Warren^4^). The London gin epidemic from 1690 to 1752 led to a petition by the London College of Physicians to the House of Commons to reimpose a tax on spirits, noting “Spirituous Liquors…[are] too often the cause of weak, feeble, and distempered children who must be instead of an advantage and strength a charge to their country.”^4^ Their petition implicated distilled spirits, rather than alcohol, per se, and did not impugn beer. Human and animal studies from the early 20th century suggested that PAE adversely affected pregnancy outcomes; however, when NIAAA was first established, the prevailing view was that alcohol was not harmful to the developing fetus, and high-dose, intravenous alcohol continued to be administered to some pregnant women to prevent premature labor. Thus, one of NIAAA’s seminal accomplishments was the nurturing of FASD research and the deployment of research findings to alert clinicians, legislators, and the public to the dangers of PAE.

This brief review focuses on selected discoveries of the last half century on the effects of PAE, highlighting the work of NIAAA-funded researchers as well as the Collaborative Initiative on Fetal Alcohol Spectrum Disorders (CIFASD), a research consortium funded by NIAAA from 2003 until the present, for which Dr. Ed Riley has served as principal investigator and the author of this review has served as scientific director. Readers are referred to more comprehensive reviews of FASD for additional information.^5^,^6^

## Fetal Alcohol Syndrome

The major functional disabilities associated with PAE are due to lifelong cognitive and behavioral impairment.^5^ Alcohol affects brain development throughout pregnancy, yet the neuropathology is often microscopic and not evident on clinical imaging. What made FAS recognizable to early investigators was not a specific neurodevelopmental syndrome, but rather the associated constellation of prenatal and postnatal growth retardation, small head circumference (microcephaly), and facial and nonfacial dysmorphology in infants or children with PAE.^2^,^3^ Microcephaly and prenatal and postnatal growth retardation are found in numerous neurodevelopmental disorders. However, alcohol exposure during one of the earliest embryonic developmental stages (i.e., gastrulation) induces relatively specific facial dysmorphology that serves as a visible marker for the underlying brain and neurodevelopmental abnormalities that cause functional impairment. This specific facial dysmorphology provided the long-missing link between PAE, abnormal brain development, and neurodevelopmental abnormalities. It is the frequent absence of this specific facial dysmorphology and the difficulty of obtaining a history of PAE that have challenged clinicians and investigators in fully characterizing the neurodevelopmental outcomes associated with alcohol exposure at other stages of gestation.

## Diagnosis of FAS and FASD

There is no biological marker or gold standard that identifies a child with FASD. Consequently, as research on FASD progressed over the past half century, diagnostic criteria for FASD, including FAS, evolved and diverged, both within the United States and in other countries.^5^,^7^–^10^

All diagnostic systems for FAS require either two or three of three cardinal facial features: short palpebral fissures; smooth nasal philtrum; and thin upper lip vermilion. All diagnostic systems also require structural and/or functional abnormalities of the central nervous system. Prenatal and postnatal growth retardation, although predictive of adverse neurodevelopmental outcomes,^11^ are not universally required for diagnosis. FAS may be diagnosed in the absence of a history of PAE, given the relative specificity of the cardinal facial features, particularly after ruling out phenocopies of FAS, including genetic conditions and other teratogenic exposures (see Table 4 in Hoyme et al. [2016]^7^).

Absent a gold standard, no diagnostic system can be considered superior, and agreement among diagnostic systems within a single cohort is modest.^8^,^9^ Clearly, clinical care and research on FASD would benefit from the harmonization of these various diagnostic and classification systems. Below is a more detailed description of one representative diagnostic framework, which was developed by the Collaboration on Fetal Alcohol Spectrum Disorders Prevalence (CoFASP), a study funded by NIAAA to investigate the epidemiology of FASD across the United States.^7^ According to CoFASP, the umbrella term FASD encompasses any one of four conditions: FAS, partial fetal alcohol syndrome (PFAS), alcohol-related neurodevelopmental disorder (ARND), and alcohol-related birth defects (ARBD) (see Table 1).^7^

The CoFASP diagnostic criteria for FAS require abnormalities in four clinical domains: craniofacial anomalies; growth retardation; abnormal brain structure or function; and neurobehavioral impairment.^7^ Short palpebral fissures are identified when direct measures are in the 10th centile or below. Smooth philtrum and thin vermilion border are identified by comparing facial features to racially normed lip/ philtrum charts. Only two of the three cardinal craniofacial anomalies need be present for an FAS diagnosis. Prenatal and/ or postnatal growth deficiency is defined as height and/or weight in the 10th centile or below. Abnormal brain structure or function may include head circumference in the 10th centile or below, structural brain abnormalities, or recurrent nonfebrile seizures.

In the CoFASP framework, neurobehavioral impairment is measured using standardized tests and may include cognitive deficits, such as low full-scale IQ, as well as impairment of executive functioning, learning, memory, visuospatial perception, or behavior, including self-regulation, attention, and impulse control. Most of these impairments are defined based on scores of at least 1.5 standard deviation (SD) below the mean. For children younger than age 3, the criterion for neurobehavioral impairment is met if there is developmental delay of at least 1.5 SD below the mean.^7^ Other diagnostic systems set different thresholds for dysmorphology and neurobehavioral impairment. For example, a framework included in the most recent edition of the *Diagnostic and Statistical Manual of Mental Disorders* (DSM-5)^12^ as a condition for further study focuses on three domains of impairment (i.e., neurocognitive, self-regulation, and adaptive function).^13^ Mattson and colleagues provide a detailed description of neurobehavioral impairment in FASD.^14^

According to the CoFASP framework, with documented PAE, a diagnosis of PFAS is made when there is cardinal facial dysmorphology and neurobehavioral impairment but no growth retardation and no abnormal brain structure or function; absent evidence of PAE, the diagnosis of PFAS additionally requires growth deficiency or deficient brain growth.^7^ ARND is diagnosed when there is neurobehavioral impairment and a history of PAE but no cardinal facial dysmorphology.^7^ Although many children with FASD have a constellation of dysmorphic features affecting the face, limbs, and internal organs, PAE in rare cases causes major malformations without neurobehavioral impairment, structural brain abnormalities, or growth retardation. This condition is referred to as ARBD.^7^

The definition of PAE assumes great importance in clinical diagnosis and research but differs among the different diagnostic systems. CoFASP defines PAE based on one of the followed criteria for maternal alcohol consumption: six or more drinks per week for two or more weeks during pregnancy; three or more drinks on at least two occasions; alcohol-related social or legal problems around the time of pregnancy; documented intoxication during pregnancy; positive testing for biomarkers associated with alcohol exposure; or a positive screen using a validated tool for alcohol use.^7^

## Alcohol Is a Teratogen

Many children diagnosed with FASD have been exposed to other drugs, such as nicotine, cannabinoids, opioids, or stimulants; have nutritional deficiency; are raised in chaotic households; and experience numerous adverse childhood events. Separately, each of these insults may cause neurobehavioral impairment. Therefore, not surprisingly, there was initial reluctance to accept that alcohol causes birth defects (i.e., is a teratogen).

Animal models can control for many of these confounding variables and provided the first strong evidence that alcohol was indeed teratogenic. Sulik and colleagues showed that a single alcohol exposure during gastrulation in mice caused microcephaly, growth retardation, and the cardinal facial features of FAS in the absence of nutritional deficiency or other teratogens.^15^ This discovery drew a direct connection between alcohol and the constellation of developmental abnormalities described less than a decade earlier in humans with FAS. It allowed the conclusion that alcohol toxicity causes FAS, even though concurrent teratogenic exposures, genetic polymorphisms, nutritional deficiency, and stressors may further impact craniofacial and brain development. Because gastrulation occurs during the third week of human gestation, when many women are unaware of their pregnancies, this seminal work also underscored the potential for binge drinking to cause FAS prior to pregnancy recognition.

Later work from the same laboratory highlighted both the relative specificity and insensitivity of the cardinal facial dysmorphology of FAS as a marker of PAE.^16^,^17^ Whereas alcohol exposure in mice on gestational day 7 (corresponding to gastrulation) reproduced the cardinal facial dysmorphology of FAS, exposure on gestational day 8.5 (corresponding to neurulation) produced different facial anomalies more characteristic of DiGeorge syndrome or retinoic acid embryopathy. Indeed, retinoic acid exposure and alcohol exposure during gastrulation in mice caused similar malformations. Although alcohol and retinoic acid are chemically unrelated, their common potentiation of programmed cell death in selected embryonic cell populations induced similar, stage-dependent, developmental outcomes.^18^,^19^

These animal studies demonstrated that the presence and pattern of craniofacial malformations were dependent on the timing of teratogen exposure. The cardinal facial dysmorphology that was first and irrevocably associated with FAS proved to be a happenstance of alcohol exposure during gastrulation. These discoveries contributed to the recognition that, at least in some people, neurobehavioral impairment due to PAE could occur in the absence of cardinal facial dysmorphology or any facial dysmorphology at all, as is the case in people with ARND.

## The Face Is a Window to the Brain

The first descriptions of FAS identified a variety of craniofacial abnormalities in addition to the cardinal features of short palpebral fissures, smooth nasal philtrum, and thin upper lip vermilion. Some of these malformations, such as maxillary hypoplasia, ptosis, and retrognathia, occur in a host of developmental disorders and are readily recognized by geneticists, dysmorphologists, and developmental pediatricians. Therefore, a major quest for the field has been the discovery of other patterns of facial or nonfacial dysmorphology that might also link neurobehavioral impairment to PAE, even in the absence of a history of PAE.

An equally important goal has been to simplify or automate the detection of any defining facial dysmorphology to facilitate diagnosis for the many patients that lack access to highly specialized clinicians. Astley used computer analysis of two-dimensional facial photographs to evaluate the diagnostic features defined by the FASD 4-Digit Diagnostic Code,^20^ whereas CIFASD and other investigators employed automated analysis of three-dimensional (3D) facial images. Suttie and colleagues used dense surface modeling to study facial dysmorphology in 3D images of children from the CIFASD cohort.^21^ This method allowed them to quantitate facial shape and to sort facial images based on the degree to which they resembled those of children with FAS or controls. This analysis differentiated children with PAE whose faces did not clearly show the characteristic features (i.e., those who had nonsyndromal faces) from children without PAE with greater than 90% specificity. Importantly, children with PAE who had nonsyndromal facial features also had significantly lower IQ and learning ability than children whose faces more closely resembled controls. Using dense surface modeling, Muggli and colleagues demonstrated that even mild PAE could affect facial shape.^22^

Technology has evolved to enable the acquisition of 3D images on smartphones, and contour analysis can be automated in the Cloud. Hence, it may be possible to automate the analysis of facial dysmorphology and facilitate the diagnosis of FASD wherever access to internet-connected smartphones is available.

## Epidemiology of FASD

FASD is the most common preventable cause of intellectual disability.^23^ Using active case ascertainment, CoFASP investigators estimated the prevalence of FASD among first-grade students to be 1% to 5% across four regions of the United States.^24^ These conservative estimates of FASD prevalence equal or exceed those for autism spectrum disorder. Among 222 cases identified as FASD within this cohort, 12% were classified as FAS, 47% as PFAS, and 41% as ARND. However, only two of the 222 children (1%) had previously been diagnosed with FASD, highlighting the extent to which FASD is underrecognized or misdiagnosed.^25^

Estimates of FASD prevalence vary across studies, in part because of differences in study methodology and classification definitions. One meta-analysis estimated the global prevalence of FAS at 0.15% and FASD at 0.77%.^26^ Prevalence estimates also vary across different countries due to cultural differences in drinking. One of the highest estimates of FASD prevalence has been 14% to 21% in the wine-growing region of the Western Cape Province of South Africa, where weekend binge drinking has been common.^27^

High rates of binge drinking during the childbearing years are an important contributor to the high prevalence of FASD in the United States. Approximately 25% of Americans ages 18 to 44 binge drink, 45% of pregnancies are unintended, and gastrulation often occurs before a woman is aware of her pregnancy.^28^,^29^ Among pregnant women, the prevalence of any alcohol use (10%) and binge drinking (3%) within the past 30 days is also high. The combination of binge drinking and sex without contraception greatly increases the risk of an alcohol-exposed pregnancy.

Whereas binge drinking is a widely accepted risk for FASD, there is less certainty regarding the risk associated with low or moderate levels of alcohol consumption during pregnancy, stemming in part from the inherent challenge of proving safety as opposed to harm. Both human and animal studies have failed to establish a threshold for safe drinking during pregnancy.^30^ For example, in cell culture experiments, alcohol concentrations corresponding to those achieved in the blood and fetus after just one drink inhibit cell adhesion mediated by the developmentally critical L1 neural cell adhesion molecule.^31^ In humans, intake of less than five to six standard U.S. drinks per week is associated with craniofacial dysmorphology and neurobehavioral impairment.^22^,^30^,^32^ Research funded by NIAAA has played a major role in informing the advisories from the U.S. Surgeon General that women who are pregnant or trying to conceive should not consume alcoholic beverages.^4^

## The Neurodevelopmental Effects of PAE

Early autopsy studies in infants and children with FAS revealed major brain malformations.^33^ Among these were microcephaly, agenesis or hypoplasia of the corpus callosum, ventricular enlargement, dysplasia of the anterior lobes of the cerebellum, and neuronoglial heterotopias—findings consistent with major disruption of neurogenesis, neural cell migration, and the premature triggering of programmed cell death. These gross neuropathological abnormalities are not observed in most children with FASD but highlight the mechanisms underlying similar, but milder, abnormalities in grey matter thickness, microstructural white matter abnormalities, decreased brain volume, and neuronal and glial migration defects.^5^ Prenatal alcohol exposure also alters the trajectory of grey matter development during childhood.^34^ In some studies, facial abnormalities correlated with volume reductions in specific brain regions, reinforcing the concept that face and brain dysmorphology arise concurrently and that the face is a window to the brain.^17^,^35^,^36^ Clinical imaging in children with FASD is frequently normal, reflecting the microscopic nature of brain developmental abnormalities that underlie typical neurobehavioral impairments related to PAE. Overall, studies found that neurodevelopmental outcomes are related to the quantity, frequency, and timing of alcohol exposure as well as to maternal age, nutritional status, socioeconomic status, and genetic background of both mother and fetus.^5^

Animal studies have shown that alcohol disrupts brain development through a variety of mechanisms. Alcohol causes oxidative injury and programmed cell death in neural crest cells destined to form craniofacial and brain structures.^15^,^18^,^37^ Alcohol is metabolized to acetaldehyde, a toxic molecule that chemically modifies and damages DNA and cells.^38^ Alcohol also produces enduring epigenetic changes^39^ that alter DNA transcription and diverse signaling pathways involved in brain development. Moreover, alcohol impairs neurogenesis and diverts differentiation of neural stem cells from neural to nonneural lineages, contributing to brain volume reductions.^38^ Early research further identified similarities between FAS and milder phenotypes of syndromes associated with holoprosencephaly,^40^ a disorder that affects midline craniofacial and brain development and is sometimes associated with mutations in the Sonic hedgehog (Shh) gene. Alcohol similarly disrupts the Shh signaling pathway,^41^,^42^ thereby altering the function of primary cilia^43^—cellular organelles that are critical for development.

Alcohol also may disrupt neuronal cell migration and synaptic connections through its interactions with the L1 protein, a developmentally critical neural cell adhesion molecule that guides neuronal cell migration and axon pathfinding. Alcohol inhibits L1-mediated cell adhesion at half maximal concentrations achieved after just one drink.^31^ Alcohol blocks L1 adhesion by binding to specific amino acids that regulate the interaction of L1 molecules located on adjacent cells.^44^ The nanopeptide NAPVSIPQ potently antagonizes alcohol inhibition of L1 adhesion and prevents alcohol teratogenesis in mouse embryos.^45^

Finally, genetic factors also may influence the development of FAS and alcohol’s effects on neurodevelopment. Concordance for FAS is higher in monozygotic than dizygotic twins,^46^ and diverse genes have been identified that modulate the effects of alcohol on craniofacial and brain development.^47^,^48^

## Biomarkers of Alcohol Exposure and Adverse Outcome Risk

In many cases, information on an infant’s history of PAE is unavailable or unreliable, hampering the clinical diagnosis of FASD and related research. Analyses of early markers of alcohol exposure, such as fatty acid ethyl esters in meconium, can provide relatively sensitive and specific confirmation of PAE in the last two trimesters of pregnancy^49^ but are not routinely performed in clinical practice. More recent research from CIFASD has raised hopes that biomarkers of exposure and risk for adverse outcomes may be obtained during the second trimester to identify infants and children requiring early intervention. For example, maternal blood samples from the second trimester of pregnancy showed increased methylation of pro-opiomelanocortin and period 2 genes,^50^ unique cytokine signatures,^51^ and a unique profile of micro RNAs linked to alcohol exposure and neurodevelopmental delay.^52^ Infant plasma micro RNAs also predicted PAE-associated growth restriction and cognitive development.^53^

Some of these biomarkers also may be mediators of biological effects of PAE. Decreased expression of proopiomelanocortin was associated with increased levels of cortisol in children with PAE, consistent with disinhibition of the hypothalamic pituitary stress axis.^50^ The identified micro RNAs were shown to collectively modulate placental growth and development,^54^ and proinflammatory cytokines may predispose to autoimmune and inflammatory conditions later in life.^55^,^56^

## FASD Across the Lifespan

The effects of PAE on morphology and neurobehavior and health are lifelong.^5^ As children with FASD mature into adulthood, the cardinal facial dysmorphology may become less pronounced, making diagnosis in adulthood more difficult.^57^ More challenging still is the diagnosis of FASD in adults with neurobehavioral disorders who lack both cardinal facial dysmorphology and a history of PAE. The high prevalence of FASD makes it likely that many such individuals are followed in adult medical practices without ever being diagnosed.

A growing area of FASD research concerns the developmental origins of health and disease associated with PAE.^58^ Premature death and increased prevalence of metabolic, immune, and cardiovascular disorders have been reported in informal surveys of adults with FASD^56^ as well as in epidemiological studies.^55^,^59^ For example, studies in human cohorts and zebrafish indicate that PAE induces elements of metabolic syndrome in adults by modifying developmental programs for hepatic and adipose tissue embryogenesis.^60^ Further research will be important to delineate the full range of human diseases associated with PAE to allow for earlier detection and intervention.

## The Next 50 Years

What should we hope for from the next 50 years of NIAAA-funded research on FASD? There will never be enough specialized clinics to diagnose and treat the large numbers of children and adults with FASD. Recent advances in remote diagnosis of facial dysmorphology and in neurobehavioral assessment^61^ hold promise for broader access to automated, cloud-based screening and diagnostic tools. The identification of more specific markers of PAE and adverse developmental outcomes will greatly aid diagnosis. Treatment is similarly limited by the high prevalence of FASD in relation to the availability of skilled therapists. The refinement of early interventions and their translation to accessible online platforms will be necessary to fully address the public health burden of FASD. App-based approaches show early promise but still require considerable development and refinement.^62^ Studies on the postnatal administration of choline to mitigate the neurodevelopmental effects of PAE also have been encouraging.^63^–^65^ Finally, the high prevalence of FASD will most readily be reduced by continued progress in one of NIAAA’s primary missions—the development of effective strategies to prevent and treat alcohol use disorder and the patterns of drinking that engender PAE. Equally important will be the reduction in stigma associated with these disorders, so that effective strategies are embraced by those at risk or affected.

## Figures and Tables

**Table 1:** Diagnostic Criteria for Four Conditions Within the FASD Spectrum According to CoFASP.^5^

Diagnostic Criterium	FAS		Partial FAS		ARND	ARBD
Confirmed Prenatal Alcohol Exposure^a^	Yes	No	Yes	No	Yes	Yes
Facial Dysmorphology^b^	Required	Required	Required	Required	Not required	N/A
Growth Deficiency^c^	Required	Required	Not required	Required if brain abnormality is not present	Not required	N/A
Brain Abnormality^d^	Required	Required	Not required	Required if growth deficiency is not present	Not required	N/A
Cognitive or Behavioral Impairment^e^	Required	Required	Required	Required	Required*	N/A
Other Systemic Malformation	Not required	Not required	Not required	Not required	Not required	Required

a Defined as ≥ 6 drinks/week for 2 weeks or ≥ 3 drinks on ≥ 2 occasions; documentation of maternal intoxication in records; positive biomarker for alcohol; or evidence of risky maternal drinking on a validated screening tool.

b Defined as ≥ 2 of the following: short palpebral fissures, thin vermilion border, and smooth philtrum.

c Defined as height and/or weight ≤ 10^th^ centile based on racially/ethnically normed charts.

d Defined as head circumference ≤ 10^th^ centile, structural brain anomaly, or recurrent nonfebrile seizures.

e Cognitive impairment is defined as global cognitive impairment, verbal or spatial IQ, or individual neurocognitive domain ≥ 1.5 SD below mean. Behavioral impairment is defined as impairment of self-regulation ≥ 1.5 SD below mean. For children under age 3, developmental delay is required.

* ARND requires two behavioral or cognitive deficits if IQ is not ≥ 1.5 SD below the mean.

*Note:* ARBD, alcohol-related birth defects; ARND, alcohol-related neurodevelopmental disorder; FAS, fetal alcohol syndrome; N/A, not applicable.

*Source:* Adapted with permission from Wozniak et al.^5^
